# The risk factors and nursing countermeasures of sepsis after cesarean section: a retrospective analysis

**DOI:** 10.1186/s12884-022-04982-8

**Published:** 2022-09-09

**Authors:** Meiniang Shi, Lanlan Chen, Xiaoyun Ma, Biyu Wu

**Affiliations:** Department of Nursing, Quanzhou First Hospital, No. 248 East Street, Quanzhou City, 362002 Fujian Province China

**Keywords:** Sepsis, Cesarean section, Care, Prevention, Management

## Abstract

**Background:**

Sepsis is a very serious complication of cesarean section, understanding the influencing factors is important to the prevention and management of sepsis. We aimed to analyze the associated risk factors of sepsis of cesarean section, to provide evidences into the clinical management and nursing care of cesarean section.

**Methods:**

Patients who underwent cesarean section surgery from January 1, 2017 to June 30, 2021 in our hospital were included. The characteristics of patients were collected and analyzed. Logistic regression analyses were conducted to analyze the influencing factors of sepsis of cesarean section.

**Results:**

A total of 3819 patients undergoing cesarean section were included, the incidence of sepsis in patients undergoing cesarean section was 0.84%. There were significant differences in the age, vaginal delivery attempt, premature rupture of membranes, preoperative hemoglobin, estimated blood loss during surgery and postoperative urinary tube implacement between sepsis and no sepsis patients (all *p* < 0.05). Logistic regression analyses found that age ≥ 35y(OR3.22, 95%CI1.20 ~ 5.15), gestational diabetes(OR2.64, 95%CI1.91 ~ 4.15), vaginal delivery attempt(OR2.05, 95%CI1.70 ~ 4.42), premature rupture of membranes(OR2.42, 95%CI1.02 ~ 4.20), preoperative hemoglobin ≤ 105 g/L(OR4.39, 95%CI1.02 ~ 7.88), estimated blood loss during surgery ≥ 400 ml(OR1.81, 95%CI1.35 ~ 3.01), postoperative urinary tube implacement(OR2.19, 95%CI1.27 ~ 2.50) were the risk factors of sepsis in patients undergoing cesarean section(all *p* < 0.05). Escherichia Coli(46.15%), Enterococcus faecalis(17.95%) and Pseudomonas aeruginosa(12.83%) were the most commonly-seen bacteria in sepsis patients.

**Conclusion:**

In clinical practice, medical workers should carry out strict management and early prevention of related risk factors during the perioperative period of pregnant women, to effectively reduce the occurrence of sepsis after cesarean section.

## Background

In the past two decades, with the change of people's concept of fertility and the improvement of fetal monitoring, the cesarean section rate has been on the rise globally [1]. Previous surveys [2, 3] have found that the cesarean section rate in China is as high as 46. 5%. Cesarean section surgery is a double-edged sword with some risk of adverse outcomes, and postoperative infection is one of the most common complications after cesarean section surgery. Although antibiotics have been widely used to prevent postpartum infection of cesarean section, the risk of abdominal incision infection, uterine incision infection, endometritis and urinary tract infection after surgery is still increasing [4]. In severe cases, sepsis, postpartum hemorrhage, shock and even life-threatening of patients may occur [5]. Therefore, how to prevent infection after cesarean section has become a common concern of all obstetricians.

Recent studies have shown that the rate of maternal death caused by postpartum sepsis in developing countries accounts for about 10.7%, only second to postpartum hemorrhage [6]. Sepsis is a clinical syndrome that results from the dysregulated inflammatory response to infection that leads to organ dysfunction [7]. Cesarean section increases the risk of postoperative infection, which can then develop into sepsis. The results of many previous studies [8–10] have shown that the occurrence of postoperative sepsis can significantly increase the risk of death in patients. The early prevention and treatment of postoperative sepsis is of great significance to improve the prognosis of patients. Although the risk factors for sepsis after cesarean delivery already well-known, there can be some risk factors that have not been fully elucidated. Besides, the incidence of sepsis after cesarean delivery is not very high in clinical setting, the risk factors for sepsis should be investigated from different population in different areas. Therefore, this study aimed to explore the risk factors of sepsis after caesarean section, to provide references for clinical prevention and treatment of postoperative sepsis.

## Methods

### Ethical consideration

In this study, all methods were performed in accordance with the relevant guidelines and regulations. This present study was a retrospective study design, the study protocol had been discussed and approved by the ethical committee of our hospital (approval number:170068). And written informed consents had been obtained from all the included patients.

### Calculation of sample size

The sample size for analysis was calculated by the comparison formula of two groups [11]: $n=\frac{\lambda }{{2\left(arcsin\sqrt{{P}_{max}}-arcsin\sqrt{{P}_{min}}\right)}^{2}}$, we presumed that α = 0.05, β = 0.2, ν = 3 − 1 = 2, then the λ = 8.84, set Pmax, Pmin, respectively, as the reported maximum and minimum incidence of sepsis after cesarean Sect. (0.48% and 1.12%) [12, 13], then it come to the results that n≈26. Therefore, at least 26 sepsis cases should be included.

### Patients

This study selected patients who underwent cesarean section surgery from January 1, 2017 to April 30, 2021 in the obstetrics department of our hospital as the research population. The inclusion criteria of patients were: adult patients aged ≥ 18 years old; patient underwent cesarean section treatment in our hospital; clinical data of the patient were complete; patient was well-informed and agreed to participate in this study. Patients with unknown or unavailable clinical data, disagreed to participant in this study were excluded.

### Diagnosis of sepsis

The diagnosis of sepsis referred to the Chinese guidelines for diagnosis and treatment of sepsis [14, 15]. The diagnosis of sepsis must meet at least the following 2 items or more: (1) The patient has symptoms of infection, including white blood cell count > 12*10^9^, body temperature > 38 ℃, respiratory rate > 20 beats/min, heart rate > 90 beats/min. (2) Infection or systemic inflammatory response syndrome appeared. (3) Signs of organ failure or tissue hypoperfusion. (4) hypotension (despite adequate fluid resuscitation) and abnormal perfusion.

### Data collection

The two authors collected patient-related clinical data, including age, body mass index (BMI), gestational age, history of miscarriage, gestational diabetes, gestational hypertension, vaginal delivery attempt, premature rupture of membranes, preoperative white blood cells, preoperative red blood cells value, preoperative hemoglobin, duration of surgery, estimated blood loss during surgery, postoperative urinary tube implacement.

### Statistical methods

We used SPSS 22.0 statistical software for data analysis. Count data were expressed as percentage (%), and the chi-square test was used for comparison between groups. Measurement data were expressed as mean ± standard deviation, and comparison between groups was performed by t test. We used logistic regression to calculate odds ratio (OR) and its 95% confidence interval (CI). In this study, the difference was statistically significant with *p* < 0.05.

## Results

### The characteristics of patients with cesarean section

All the included patients were agreed to participant in this study, and only two patients were excluded for missing data. Finally, a total of 3819 patients undergoing cesarean section in our hospital were included, of whom 32 patients had sepsis, the incidence of sepsis in patients undergoing cesarean section was 0.84%. As presented in Table 1, there were significant differences in the age, vaginal delivery attempt, premature rupture of membranes, preoperative hemoglobin, estimated blood loss during surgery and postoperative urinary tube implacement between sepsis and no sepsis patients (all *p* < 0.05). No significant differences in the BMI, gestational age, history of miscarriage, gestational hypertension, preoperative white blood cells, preoperative red blood cells value, duration of surgery were found between sepsis and no sepsis patients (all *p* > 0.05).

**Table 1:** The characteristics of included patients

Items	Sepsis group(*n* = 32)	No sepsis group(*n* = 3787)	p
Age(y)	36.54 ± 2.02	32.69 ± 2.61	0.034
BMI(kg/m^2^)	27.14 ± 3.17	27.02 ± 2.44	0.069
Gestational age(w)	38.53 ± 3.06	38.29 ± 3.15	0.102
History of miscarriage	2(6.25%)	208(5.49%)	0.059
Gestational diabetes	19(59.38%)	406(10.72%)	0.006
Gestational hypertension	9(28.13%)	915(24.16%)	0.075
Vaginal delivery attempt	11(34.28%)	201(5.31%)	0.012
Premature rupture of membranes	28(87.50%)	268(7.08%)	0.027
Preoperative white blood cells (× 10^9^/L)	9.42 ± 3.16	8.55 ± 3.98	0.109
Preoperative red blood cells value(× 10^12^/L)	4.18 ± 1.67	4.22 ± 2.01	0.084
Preoperative hemoglobin (g/L)	96.12 ± 47.31	125.07 ± 44.52	0.025
Duration of surgery(min)	23.09 ± 9.22	23.15 ± 8.91	0.081
Estimated blood loss during surgery(ml)	528.19 ± 104.72	202.78 ± 59.23	0.001
Postoperative urinary tube implacement	27(84.38%)	1014(26.78%)	0.002

### Risk factors of sepsis in patients undergoing cesarean section

The variable assignment of multivariate logistic regression was showed in Table 2. We had found significant differences in the incidence of sepsis in patients with different age, gestational diabetes, vaginal delivery attempt, premature rupture of membranes, preoperative hemoglobin, estimated blood loss during surgery, postoperative urinary tube implacement (all *p* < 0.05). As indicated in Table 3, logistic regression analyses found that age ≥ 35y(OR3.22, 95%CI1.20 ~ 5.15), gestational diabetes(OR2.64, 95%CI1.91 ~ 4.15), vaginal delivery attempt(OR2.05, 95%CI1.70 ~ 4.42), premature rupture of membranes(OR2.42, 95%CI1.02 ~ 4.20), preoperative hemoglobin ≤ 105 g/L(OR4.39, 95%CI1.02 ~ 7.88), estimated blood loss during surgery ≥ 400 ml(OR1.81, 95%CI1.35 ~ 3.01), postoperative urinary tube implacement(OR2.19, 95%CI1.27 ~ 2.50) were the risk factors of sepsis in patients undergoing cesarean section(all *p* < 0.05).

**Table 2:** The variable assignment of multivariate logistic regression

Factors	Variables	Assignment
Sepsis	Y	yes = 1, no = 2
Age(y)	X_1_	≥ 35 = 1, < 35 = 2
Gestational diabetes	X_2_	yes = 1, no = 2
Vaginal delivery attempt	X_3_	yes = 1, no = 2
Premature rupture of membranes	X_4_	yes = 1, no = 2
Preoperative hemoglobin (g/L)	X_5_	≤ 105 = 1, > 105 = 2
Estimated blood loss during surgery(ml)	X_6_	≥ 400 = 1, < 400 = 2
Postoperative urinary tube implacement	X_7_	yes = 1, no = 2

**Table 3:** Logistic regression analyses on the risk factors of sepsis in patients undergoing cesarean section

Variables	Unadjusted		Adjusted		p
	OR	95%CI	OR	95%CI	
Age ≥ 35y	5.08	3.81 ~ 6.11	3.22	1.20 ~ 5.15	0.011
Gestational diabetes	3.36	2.13 ~ 5.92	2.64	1.91 ~ 4.15	0.007
Vaginal delivery attempt	3.44	2.01 ~ 5.17	2.05	1.69 ~ 4.42	0.026
Premature rupture of membranes	3.18	1.79 ~ 4.48	2.42	1.02 ~ 4.20	0.042
Preoperative hemoglobin ≤ 105 g/L	6.32	5.14 ~ 8.22	4.39	1.02 ~ 7.88	0.017
Estimated blood loss during surgery ≥ 400 ml	2.95	1.77 ~ 3.96	1.81	1.35 ~ 3.01	0.009
Postoperative urinary tube implacement	3.04	2.14 ~ 4.67	2.19	1.27 ~ 2.50	0.047

### Pathogen distributions

Of the 32 sepsis patients, a total of 39 cases of pathogens were detected from the blood specimens. As presented in Table 4, Escherichia Coli(46.15%), Enterococcus faecalis(17.95%) and Pseudomonas aeruginosa(12.83%) were the most commonly-seen bacteria in sepsis patients.

**Table 4:** The pathogen distributions of patients with sepsis

Pathogens	Cases	Percent
Gram-positive bacteria	11	28.21%
Enterococcus faecalis	7	17.95%
Streptococcus pharyngitis	3	7.69%
Staphylococcus haemolyticus	1	2.56%
Gram-negative bacteria	28	71.79%
Escherichia Coli	18	46.15%
Pseudomonas aeruginosa	5	12.83%
Klebsiella pneumoniae	2	5.13%
Acinetobacter baumannii	2	5.13%
Enterobacter cloacae	1	2.56%
Total	39	100%

## Discussion

Previous studies [16, 17] have shown that there are many high-risk factors that affect infection after cesarean section. Pregnant women may have basic diseases such as severe anemia, hypoproteinemia, malnutrition, diabetes, obesity, etc. [18]. Premature rupture of the membrane, especially for patients undergoing cesarean section after labor, has prolonged labor, multiple vaginal examinations and anal examinations, and amniotic fluid contamination, etc., which reduce the patient's body resistance and easily lead to postoperative infection [19–22]. Previous studies have reported that the incidence of sepsis after cesarean section varies from 0.48% to 1.12% [12, 13]. The results of this study have showed that the incidence of sepsis in patients undergoing cesarean section is 0.84%, age ≥ 35y, gestational diabetes, vaginal delivery attempt, premature rupture of membranes, preoperative hemoglobin ≤ 105 g/L, estimated blood loss during surgery ≥ 400 ml, postoperative urinary tube implacement are the risk factors of sepsis in patients undergoing cesarean section. Besides, Escherichia Coli, Enterococcus faecalis and Pseudomonas aeruginosa are the most commonly-seen bacteria in sepsis patients. Clinical preventions and nursing cares are warranted for those patients.

Previous studies [23, 24] have shown that the risk of infection after cesarean section in pregnant women with diabetes is four times that of non-diabetic women. Due to the long-term significant reduction of body defense function of diabetic women, all stages of the bacterial response are inhibited, including the phagocytosis of neutralizing toxins, intracellular bactericidal effect and cellular immunity, etc., which makes the patient prone to infection [25–27]. Moreover, sugar and fat metabolism disorders in patients with gestational diabetes, resulting in reduced postoperative tissue repair ability, slow healing of incisions, and increased probability of bacteria entering the bloodstream [28]. Besides, hyperglycemia provides support for the reproduction and spread of bacteria [29, 30]. Therefore, clinically, hidden cases should be detected as early as possible through diabetes screening, and the blood sugar level of diabetic women and the indications of cesarean section should be strictly controlled to reduce the risk of infection [31, 32].

Due to prolonged labor, stagnant active period of the parturient, vaginal delivery attempts was transferred to cesarean section [33]. This study has found that vaginal delivery attempts is an important influencing factor of sepsis. This may be related to the fact that the vagina is connected to the outside world, and the resident bacteria are many and they are all conditional pathogens [34]. The vaginal delivery attempts make the fetal head stagnant in the vagina, and the lower part of the uterus is compressed by the fetal head for a long time, resulting in poor tissue blood supply and higher probability of infection [35, 36]. In addition, the process of vaginal trial of parturient is often mixed with excrement, and pathogenic bacteria are also easy to go up through the enlarged birth canal or into the blood through the incision of cesarean section [37]. Therefore, attention should be paid to the evaluation of prenatal delivery factors, and painless delivery techniques (such as epidurals) should be developed to minimize repeated vaginal labor attempts.

Previous studies [38, 39] have shown that the longer the catheter is indwelled, the higher the infection rate. We did not use catheters for all patients in this present study, only the patients have difficulty in urination for more than four hours got postoperative urinary tubes. Postoperative puerpera's vulva lochia and blood oozing from traumatic tissues increase the probability of contamination of the long-term indwelling catheter, which may enter the blood through urethral mucosal damage [40, 41]. Therefore, the indwelling catheter must be strictly followed and evaluated for removal. Paraffin oil or progesterone can be used as a lubricant to reduce urethral damage and remove the catheter as soon as possible. Previous studies [42, 43] have reported that the use of antibacterial drugs is an independent risk factor for patients with sepsis. Clinically, preventive medication has been given for repeated bleeding or premature rupture of membranes due to abnormal position of the placenta [44]. For patients with repeated bleeding or premature rupture of membranes, the use of antibacterial drugs will be more challenging with regards to the flora disorder and poor specificity of bacteria in those conditions [45]. Future research should further analyze the relationship between the use of antibiotics and the occurrence of sepsis.

A total of 39 strains of bacteria have been isolated in this study, mainly Gram-negative cocci, which is consistent with previous related literature reports. Streptococcus, a type of mucosal tissue symbiotic bacteria, mostly exists in the skin, mucous membranes, and female genitourinary tract. For example, Streptococcus pharyngitis is a normal genitourinary tract flora. Those with surgical trauma, diabetes, and immunocompromised are susceptible to infection and invasion [46, 47]. Therefore, cephalosporins or quinolones can be empirically selected in clinical treatment, and the use of extended-spectrum antimicrobials should be avoided. If empirical treatment is ineffective for 72 h, pathogenic evidence should be actively sought, based on the results of pathogen isolation and identification and drug susceptibility testing, and adjust the antibacterial drug treatment plan timely [48]. In view of the particularity of cesarean section, there are still some controversies about the application principles of preventive use of antibiotics in the perioperative period, such as the long-term effect of antibiotics on newborns, and for pregnant women with high-risk infection factors. The selection of narrow-spectrum or broad-spectrum antibiotics still need further research.

This study is a retrospective investigation and has some limitations. First of all, all data in this study are taken from archived medical records. Medical history data such as pregnancy and childbirth history, blood transfusion history, hospitalization history and other medical history data are provided by the parturient and cannot be fully checked for accuracy, and the investigator may have insufficiently comprehensive indicators included. Secondly, the sepsis cases in this present study are relatively small, even rough we have calculated the sample size that 32 cases are statistical power enough to detect the group differences, the results from more case number or from multi centers will be much more convincing with stronger evidences. Thirdly, the laboratory test data in this study have not involved the body's immune response, such as inflammatory factors, oxygen free radicals and other biochemical indicators, which play an important role in clarifying the mechanism of sepsis after caesarean section. Therefore, it is necessary to conduct more multi-center, multi-sample and more scientifically designed prospective studies in the future, to explore the influencing factors and related nursing strategies of sepsis after caesarean section.

## Conclusions

In summary, we have found that the incidence of sepsis in patients undergoing cesarean section is 0.84%. For patients with age ≥ 35y, gestational diabetes, vaginal delivery attempt, premature rupture of membranes, preoperative hemoglobin ≤ 105 g/L, estimated blood loss during surgery ≥ 400 ml, postoperative urinary tube implacement, they may have higher risks of postoperative sepsis. According to related risk factors, strict managements and evaluation of associated aspects of the perioperative period of pregnant women should be carried out, and early nursing interventions should be carried out to effectively prevent and reduce the incidence of sepsis after cesarean section.

## Data Availability

All data generated or analyzed during this study are included in this published article.

## Abbreviations

BMI: Body mass index
OR: Odds ratio
CI: Confidence interval

## References

[1] Sandall J, Tribe RM, Avery L, Mola G, Visser GH, Homer CS, Gibbons D, Kelly NM, Kennedy HP, Kidanto H (2018). Short-term and long-term effects of caesarean section on the health of women and children. Lancet.

[2] Liang J, Mu Y, Li X, Tang W, Wang Y, Liu Z, Huang X, Scherpbier RW, Guo S, Li M (2018). Relaxation of the one child policy and trends in caesarean section rates and birth outcomes in China between 2012 and 2016: observational study of nearly seven million health facility births. BMJ.

[3] Ming Y, Li M, Dai F, Huang R, Zhang J, Zhang L, Qin M, Zhu L, Yu H, Zhang J (2019). Dissecting the current caesarean section rate in Shanghai, China. Sci Rep.

[4] Li L, Cui H (2021). The risk factors and care measures of surgical site infection after cesarean section in China: a retrospective analysis. BMC Surg.

[5] Song C, Xu Y, Ding Y, Zhang Y, Liu N, Li L, Li Z, Du J, You H, Ma H (2021). The rates and medical necessity of cesarean delivery in China, 2012–2019: an inspiration from Jiangsu. BMC Med.

[6] Hodgetts-Morton V, Hewitt CA, Wilson A, Farmer N, Weckesser A, Dixon E, Brocklehurst P, Hardy P, Morris RK (2020). Vaginal preparation with chlorhexidine at cesarean section to reduce endometritis and prevent sepsis: A randomized pilot trial (PREPS). Acta Obstet Gynecol Scand.

[7] Devia Jaramillo G, Ibanez Pinilla M (2022). Quick Sequential Organ Failure Assessment, Sequential Organ Failure Assessment, and Procalcitonin for Early Diagnosis and Prediction of Death in Elderly Patients with Suspicion of Sepsis in the Emergency Department, Based on Sepsis-3 Definition. Gerontology.

[8] Glowicz JB (2018). Serious unintended outcomes associated with cesarean section. Am J Infect Control.

[9] Jiang HL, Lu C, Wang XX, Wang X, Zhang WY (2020). Cesarean section does not affect neonatal outcomes of pregnancies complicated with preterm premature rupture of membranes. Chin Med J (Engl).

[10] Schutte JM, Steegers EA, Santema JG, Schuitemaker NW, van Roosmalen J (2007). Maternal Mortality Committee Of The Netherlands Society Of O: Maternal deaths after elective cesarean section for breech presentation in the Netherlands. Acta Obstet Gynecol Scand.

[11] Jianwen Y, Keke L (2013). Sample size calculation methods. Statistics and Decision-Making.

[12] Dan L, Xue L, Xinyi Z (2020). Analysis of risk factors for postpartum sepsis. Chinese Journal of Advanced Education for Physicians.

[13] Zhenling W, Hua H, Ruixia G (2016). Analysis of the effect of flushing the uterine cavity with different temperatures of metronidazole solution on postpartum infection during cesarean section. Chinese Journal of Hospital Infectious Diseases.

[14] Anhua W (2014). Evidence-based guidelines for the prevention of nosocomial infections. Chinese Journal of Infection Control.

[15] Zi lin, Gua Sun, Xuli Tan: The definition, diagnostic criteria, main points and explanations of TCM syndrome diagnosis of sepsis Chinese Emergency Medicine Magazine. 2007'16(8):797–8.

[16] Foeller ME, Sie L, Foeller TM, Girsen AI, Carmichael SL, Lyell DJ, Lee HC, Gibbs RS (2020). Risk Factors for Maternal Readmission with Sepsis. Am J Perinatol.

[17] Zakosek Pipan M, Svara T, Zdovc I, Papic B, Avbersek J, Kusar D, Mrkun J (2019). Staphylococcus pseudintermedius septicemia in puppies after elective cesarean section: confirmed transmission via dam's milk. BMC Vet Res.

[18] Wankaew N, Jirapradittha J, Kiatchoosakun P (2013). Neonatal morbidity and mortality for repeated cesarean section vs. normal vaginal delivery to uncomplicated term pregnancies at Srinagarind Hospital. J Med Assoc Thai.

[19] Moudi Z, Arabnezhad L, Ansari H, Tabatabaei SM (2019). Severe maternal morbidity among women with a history of cesarean section at a tertiary referral teaching hospital in the southeast of Iran. Public Health.

[20] Acosta CD, Knight M (2013). Sepsis and maternal mortality. Curr Opin Obstet Gynecol.

[21] Bakhtawar S, Sheikh S, Qureshi R, Hoodbhoy Z, Payne B, Azam I, von Dadelszen P, Magee L (2020). Risk factors for postpartum sepsis: a nested case-control study. BMC Pregnancy Childbirth.

[22] Malmir M, Boroojerdi NA, Masoumi SZ, Parsa P: Factors affecting postpartum infection: A systematic review. Infect Disord Drug Targets. 2021;14:10–5. 10.2174/1871526521666211129100519 34844548

[23] Saeed KB, Corcoran P, Greene RA (2019). Incisional surgical site infection following cesarean section: A national retrospective cohort study. Eur J Obstet Gynecol Reprod Biol.

[24] Scholz R, Smith BA, Adams MG, Shah M, Brudner C, Datta A, Hirsch E: A Multifaceted Surgical Site Infection Prevention Bundle for Cesarean Delivery. Am J Perinatol. 2019:18(2):31–7. 10.1055/s-0039-3400993 31887748

[25] Slabuszewska-Jozwiak A, Szymanski JK, Ciebiera M, Sarecka-Hujar B, Jakiel G (2020). Pediatrics Consequences of Caesarean Section-A Systematic Review and Meta-Analysis. Int J Environ Res Public Health.

[26] Abdelraheim AR, Gomaa K, Ibrahim EM, Mohammed MM, Khalifa EM, Youssef AM, Abdelhakeem AK, Hassan H, Alghany AA, El Gelany S (2019). Intra-abdominal infection (IAI) following cesarean section: a retrospective study in a tertiary referral hospital in Egypt. BMC Pregnancy Childbirth.

[27] Zeino S, Carbillon L, Pharisien I, Tigaizin A, Benchimol M, Murtada R, Boujenah J (2017). Delivery outcomes of term pregnancy complicated by idiopathic polyhydramnios. J Gynecol Obstet Hum Reprod.

[28] Antoun L, Taweel NE, Ahmed I, Patni S, Honest H (2020). Maternal COVID-19 infection, clinical characteristics, pregnancy, and neonatal outcome: A prospective cohort study. Eur J Obstet Gynecol Reprod Biol.

[29] Leth RA, Uldbjerg N, Norgaard M, Moller JK, Thomsen RW (2011). Obesity, diabetes, and the risk of infections diagnosed in hospital and post-discharge infections after cesarean section: a prospective cohort study. Acta Obstet Gynecol Scand.

[30] Al Jama FE (2012). Risk factors for wound infection after lower segment cesarean section. Qatar Med J.

[31] Krieger Y, Walfisch A, Sheiner E (2017). Surgical site infection following cesarean deliveries: trends and risk factors. J Matern Fetal Neonatal Med.

[32] Turan O, Hakim A, Dashraath P, Jeslyn WJL, Wright A, Abdul-Kadir R (2020). Clinical characteristics, prognostic factors, and maternal and neonatal outcomes of SARS-CoV-2 infection among hospitalized pregnant women: A systematic review. Int J Gynaecol Obstet.

[33] Assaf-Balut C, Garcia de la Torre N, Duran A, Fuentes M, Bordiu E, Del Valle L, Familiar C, Valerio J, Jimenez I, Herraiz MA (2019). A Mediterranean Diet with an Enhanced Consumption of Extra Virgin Olive Oil and Pistachios Improves Pregnancy Outcomes in Women Without Gestational Diabetes Mellitus: A Sub-Analysis of the St. Carlos Gestational Diabetes Mellitus Prevention Study. Ann Nutr Metab.

[34] Mascarello KC, Horta BL, Silveira MF (2017). Maternal complications and cesarean section without indication: systematic review and meta-analysis. Rev Saude Publica.

[35] Hyldig N, Vinter CA, Kruse M, Mogensen O, Bille C, Sorensen JA, Lamont RF, Wu C, Heidemann LN, Ibsen MH (2019). Prophylactic incisional negative pressure wound therapy reduces the risk of surgical site infection after caesarean section in obese women: a pragmatic randomised clinical trial. BJOG.

[36] Shea SK, Soper DE (2019). Prevention of Cesarean Delivery Surgical Site Infections. Obstet Gynecol Surv.

[37] Wang CN, Foo J, Huang IT, Fan YC, Tsai PS, Huang CJ (2020). Identifying more risk factors for surgical site infection following cesarean section. Eur J Obstet Gynecol Reprod Biol.

[38] Yi SH, Perkins KM, Kazakova SV, Hatfield KM, Kleinbaum DG, Baggs J, Slayton RB, Jernigan JA (2019). Surgical site infection risk following cesarean deliveries covered by Medicaid or private insurance. Infect Control Hosp Epidemiol.

[39] Grabarz A, Ghesquiere L, Debarge V, Ramdane N, Delporte V, Bodart S, Deruelle P, Subtil D, Garabedian C (2021). Cesarean section complications according to degree of emergency during labour. Eur J Obstet Gynecol Reprod Biol.

[40] Chi J, Gong W, Gao Q (2021). Clinical characteristics and outcomes of pregnant women with COVID-19 and the risk of vertical transmission: a systematic review. Arch Gynecol Obstet.

[41] Mekonnen AG, Mittiku YM (2021). Surgical site infection and its association with rupture of membrane following cesarean section in Africa: a systematic review and meta-analysis of published studies. Matern Health Neonatol Perinatol.

[42] Erenberg M, Rotem R, Segal D, Yohay Z, Idan I, Yohay D, Weintraub AY: Adhesion barriers and topical hemostatic agents are risk factors for post-cesarean section infections. Surgery. 2021'20(4):7–13. 10.1016/j.surg.2021.03.048 33933281

[43] Ogah CO, Anikwe CC, Ajah LO, Ikeotuonye AC, Lawani OL, Okorochukwu BC, Ikeoha CC, Okoroafor FC (2021). Preoperative vaginal cleansing with chlorhexidine solution in preventing post-cesarean section infections in a low resource setting: A randomized controlled trial. Acta Obstet Gynecol Scand.

[44] Lee KS, Wang YL, Huang WC, Yang JH, Huang JP: Limited efficacy with additional adverse effect of anti-adhesion barrier at primary cesarean section. J Formos Med Assoc 2021;10(3):19–22. 10.1016/j.jfma.2021.03.012 33838986

[45] Smaill FM, Grivell RM: Antibiotic prophylaxis versus no prophylaxis for preventing infection after cesarean section. Cochrane Database Syst Rev. 2014;19(10):CD007482. 10.1002/14651858.CD007482.pub3 PMC8078551 25350672

[46] Lange SM, Sharpe EE, Hertzfeldt DN, Schroeder DR, Sviggum HP (2021). Effect of penicillin allergy on prophylactic antibiotic administration in the parturient undergoing cesarean delivery. Acta Anaesthesiol Scand.

[47] Baxter E (2021). A midwifery-led prevalence programme for caesarean section surgical site infections. J Hosp Infect.

[48] Weikun T (2015). Research progress in preventing infection after cesarean section. Hebei Medicine.

